# Digital Endocasting in Comparative Canine Brain Morphology

**DOI:** 10.3389/fvets.2020.565315

**Published:** 2020-10-06

**Authors:** Kálmán Czeibert, Andrea Sommese, Örs Petneházy, Tibor Csörgő, Enikő Kubinyi

**Affiliations:** ^1^Department of Ethology, Institute of Biology, ELTE Eötvös Loránd University, Budapest, Hungary; ^2^University of Kaposvár, Kaposvár, Hungary; ^3^Medicopus Nonprofit Ltd., Kaposvár, Hungary; ^4^Department of Anatomy, Cell and Developmental Biology, Institute of Biology, ELTE Eötvös Loránd University, Budapest, Hungary

**Keywords:** endocast, 3D, brain, canine, skull, CT, digital, morphology

## Abstract

Computed tomography (CT) is one of the most useful techniques for digitizing bone structures and making endocranial models from the neurocranium. The resulting digital endocasts reflect the morphology of the brain and the associated structures. Our first aim was to document the methodology behind creating detailed digital endocasts of canine skulls. We created digital endocasts of the skulls of 24 different dog breeds and 4 wild canids for visualization and teaching purposes. We used CT scanning with 0.323 mm × 0.322 mm × 0.6 mm resolution. The imaging data were segmented with 3D Slicer software and refined with Autodesk Meshmixer. Images were visualized in 3D Slicer and surface models were converted to 3D PDFs to provide easier interactive access, and 3D prints were also generated for visualization purposes. Our second aim was to analyze how skull length and width relate to the surface areas of the prepiriform rhinencephalic, prefrontal, and non-prefrontal cerebral convexity areas of the endocasts. The rhinencephalic area ratio decreased with a larger skull index. Our results open the possibility to analyze the relationship between the skull and brain morphology, and to link certain features to behavior, and cognition in dogs.

## Introduction

Endocranial casting (or endocasting) is an effective tool for studying external brain morphologies such as gyrification, sulcal pattern, olfactory bulb shape, and for making different brain measurements (e.g., distance, surface, or volume analysis), even if the brain is not accessible anymore, only the skull. In the presence of the encephalon, the magnetic resonance imaging (MRI) is preferred to the investigations due to is good resolution and distinction between the internal structures of the brain. The endocasting technique is often used in paleoanthropology to examine the encephalic morphology of extinct taxa when only the skull is available (1–4). Endocasts are usually made of latex or silicon (5, 6), which are poured into the neurocranial cavity (after closing the channels of the skull to prevent leakage). After hardening, the material is removed from the skull, and, due to the endosteal impressions, the final endocast reliably reflects the sulcal and gyral patterns of the surface of the brain. However, there are some drawbacks to using this method; for example, removing the mold can damage the fine bones of the endocranium. In order to assess the volume, the endocranium could be filled with a matrix (i.e., stones, water, or beads), but this method has proven to be imprecise and usually leads to an overestimation of the total volume (7, 8).

Currently, neuroscientists and medical personnel as well as paleontologists heavily rely on structural imaging techniques. Computed tomography (CT), which is a significant exploratory tool in biology (9, 10), is one of the most useful techniques for digitizing bone structures and making endocranial models from them (11). The resulting digital endocasts reflect the morphology of the brain (12–16) and the associated structures, such as the vascular system (17) and the cranial nerves (18). Mammals and birds are generally considered to be highly encephalized taxa (19–21), and, as their brains fill their cranial cavities, there is a strong correlation between the volume and the morphology of the endocasts and the brains in these species (20). CT provides DICOM (Digital Imaging and Communications in Medicine file format) images which can serve as a base for the creation of detailed, high-quality three-dimensional (3D) models of the osseous structures. Using this technique, it is possible to obtain digital endocasts of the endocranial space. During the later segmentation, one can decide exactly where the endosteal covering is present (thus the possible soft tissue remnants or the non-required hyperostosis can be removed digitally from a given segment). Additionally, closing the individual intracranial channels and foramina can be performed in a more precise way, as the user can clearly establish the cutting planes on the DICOM images (compared to the conventional methods which use plasticine to obliterate the foramina). This eliminates the issue of the filling material (e.g., plastic beads) leaking into irrelevant spaces (e.g., into the meatus temporalis, which is fenestrated toward the neurocranial space). When compared to the latex or silicon endocasts, the digital ones present the same reliability (7, 22) but also have additional advantages. For instance, CT does not damage the original specimen, and most of the commercial CT analysis softwares are designed to automatically calculate the volumes of the selected areas. Digital endocasts have also been used in other fields, too, such as archeological studies (23–27). 3D printing can allow these models to be materialized and replicated in any desired number, scale, and quality. CT imaging data from the entire skull have been utilized in several other studies [e.g., studying the frontal sinus anatomy (28) or the ethmoturbinate system (29), determining volume ratios of different cranial areas (30, 31), or performing finite element analysis (32)].

The amount of variation in the head shape of dog breeds is unique in the Canidae family (33). As dogs are popular pets, studying behavioral/cognitive effects of brain/skull morphology is important. In an early study, it was concluded that domestication led to behavioral changes which are probably connected to size changes in brain regions and proportions (34). This is particularly true in domestic dogs. Because skull shapes and body shapes are so diverse among breeds, dogs offer unique opportunities to observe the correlation between morphology and behavior. Data from a population of various breeds of dogs showed that the degree of gyrification of the cerebral cortex determined by the size of the brain (35). Hence, the possibility to rely on solid tools to study dogs' neuroanatomy seems more than relevant. Head shape can be measured objectively, and its common metric is the skull or cephalic index (36). Head shape is linked to behavior, brain size (37), the gross organization of the brain (38), and both the position and shape of sensory organs (39). Despite the assumption that these significant neuromorphological changes are strongly linked with behavior and health and the recent popularity of brachycephalic dogs (40), there have been few efforts to investigate the links between skull length and brain area ratios.

The first aim of the study was to document the methodology behind creating digitalized endocasts from canine skulls as, according to our knowledge, such literature is lacking despite its relevance (e.g., a potential means for veterinary education). The second aim of the study was to investigate how artificial selection for shorter or longer heads affected the prepiriform rhinencephalic, prefrontal, and non-prefrontal cerebral convexity area of the endocasts, and investigated their relationships with the skull index. We hypothesized that the endocast rhinencephalic area ratio is negatively linked to the skull index (i.e., the ratio between the maximum width and length).

## Materials and Methods

### Segmentation and Visualization

#### Subjects

Twenty-eight skulls from 24 different dog (*Canis familiaris*) breeds (Afghan hound, American bulldog, Australian shepherd, beagle, Belgian shepherd dog, border collie, borzoi, English bulldog, cane corso, chow chow, collie, English cocker spaniel, English pointer, English setter, French bulldog, German shepherd dog, Leonberger, medium German spitz, pug, Rhodesian ridgeback, saluki, Saint Bernard, Weimaraner, and Welsh terrier) and 4 wild canids [gray wolf (*Canis lupus*), coyote (*Canis latrans*), golden jackal (*Canis aureus*), and maned wolf (*Chrysocyon brachyurus*)] were selected from the collection of author TC. We chose these breeds because they represent canids with different skull sizes and shapes. Breed information was provided by the owners who donated the cadavers. The heads were removed and underwent a maceration and degreasing procedure to effectively remove all the soft and adipose tissues around and inside the bones. The exact age of each specimen was unknown, but all the skulls were from adults (confirmed based on the complete ossification of the cranial sutures and the teeth formula).

#### Imaging and Formatting

**Figure 1:**
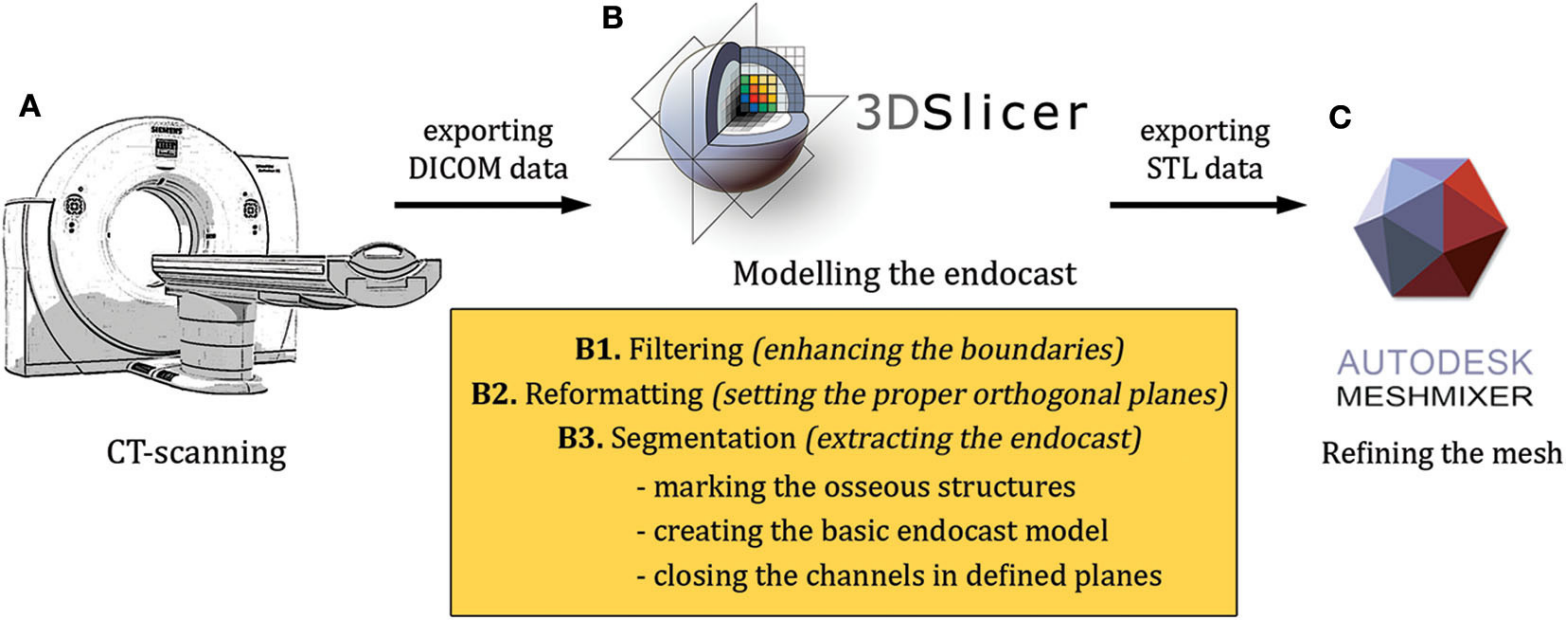
An overview of the modeling workflow from the scanning to the finalization **(A–C)**. STL, stereolithography; DICOM, Digital Imaging and Communications in Medicine file format.

The modeling included the following main steps: (1) high-resolution scanning of the skulls (medical ultra high resolution—UHR—grade); (2) creating a filter-enhanced dataset from the original image series; (3) defining and setting the main orthogonal planes; (4) segmentation of the skull; (5) segmentation of the raw endocast; (6) closure of the neurocranial channels and foramina at defined positions; (7) exporting the segmentation as a surface mesh file and refining the 3D model; (8) two- and three-dimensional visualization of the final mesh (Figure 1).

**Figure 2:**
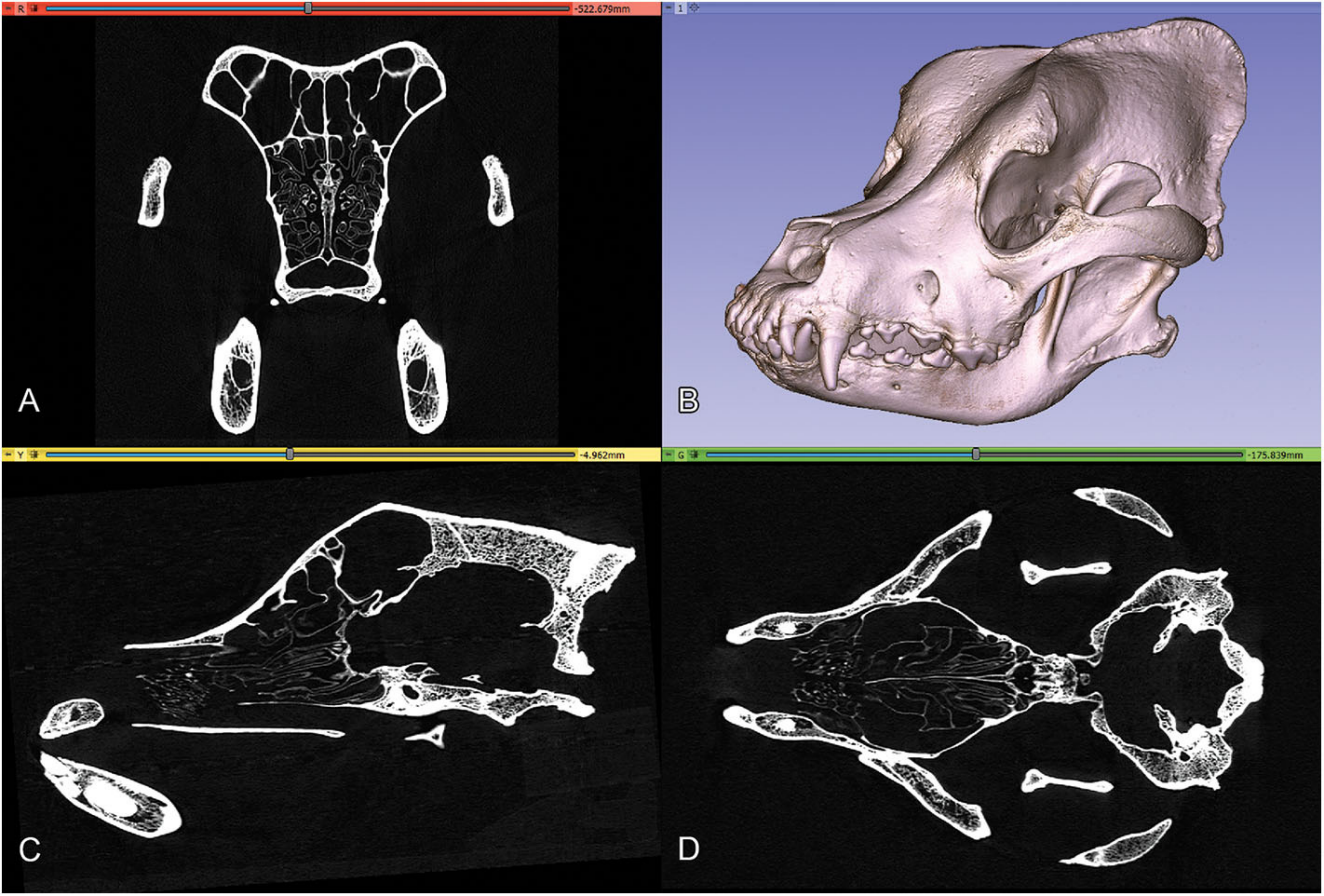
Orthogonal views and volume-rendered model of a Saint Bernard skull's CT series. **(A)** Transverse view. **(B)** 3D volume-rendered image, left rostro-lateral view. **(C)** Sagittal view. **(D)** Dorsal view.

The high-resolution imaging was performed with a Siemens Somatom Definition AS+ CT machine (Siemens, Erlangen, Germany; 170 mAs, 140 kV, pixel size 0.323 × 0.322 mm, slice thickness 0.6 mm, with a v80u bone kernel). The machine was located in Kaposvár (Hungary), at the Diagnostic and Oncoradiology Center. The raw image series were exported in a DICOM format. The DICOM images were imported into the 3D Slicer software (freeware, open source, https://www.slicer.org), where the basic DICOM volume was visualized in the orthogonal planes and a volume-rendered model from the skull was generated with the “Volume Rendering” module (Figure 2). To selectively increase the voxel density, the “*GrayscaleConnectedClosingImageFilte*r” filter was applied (which enhances the brightness for those dark areas which are surrounded by a brighter object) from the “Simple Filters” module. In this way, a new dataset was created in which the diploe of the cranial bones had a higher gray value without losing the initial detail in the endocranial contour. This helped to add the diploic channels into the skull segment during the subsequent semi-automatic segmentation process.

As a means to obtain the same segmentation planes for all the skulls, we used the “Reformat” module to precisely set the orthogonal planes: the sagittal plane was set along the midline, the dorsal plane was adjusted using the bilateral cochleae, and the spheno-occipital axis, and, finally, the transverse plane was set to be perpendicular to both the sagittal and dorsal planes.

#### Segmentation and Modeling

**Figure 3:**
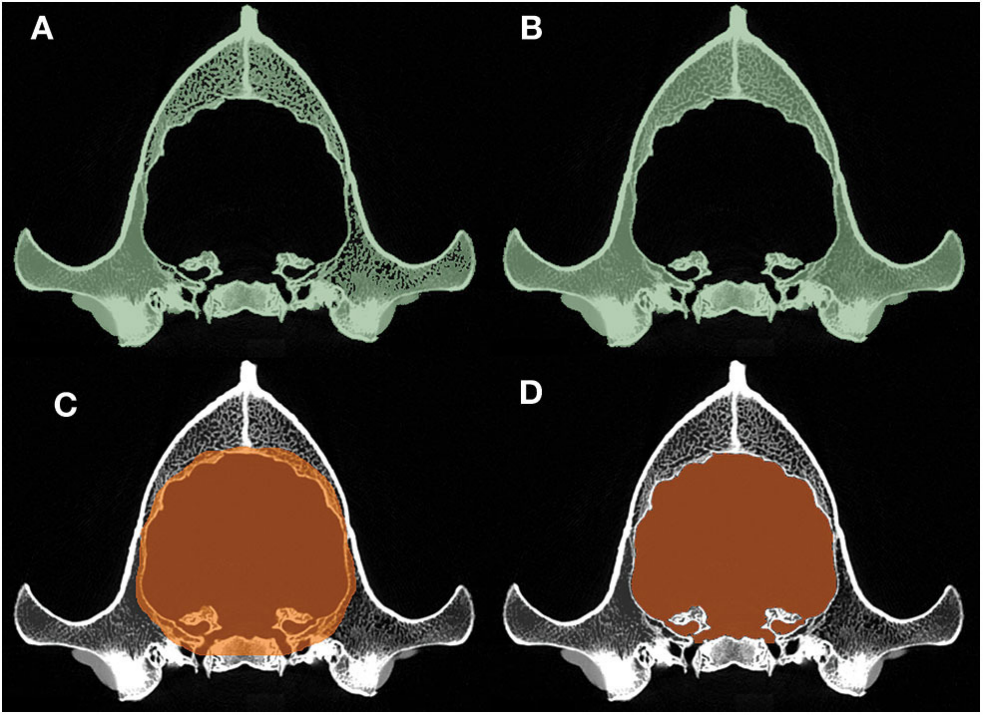
Main phases of the segmentation process. **(A)** Automatic threshold-segmentation of the skull. **(B)** Complete segmentation of the skull after filling the remaining intraosseous parts. **(C)** Highlighting the raw endocranial volume together with its neighboring osseous structures. **(D)** The final shape of the endocast after subtracting the skull segment **(B)** from the raw endocranial segment **(C)**.

Segmentation of the endocast was performed with the “Segment Editor” module of the 3D Slicer. First, a new segment of the skull was created where the threshold was adjusted to include all the osseous structures and, thus, to gain a proper lining of the intracranial cavity (Figure 3A). The remaining interlaminar holes were filled manually (Figure 3B). Afterward, a block was created which comprised the entire endocranial volume and the surrounding osseous structures (Figure 3C). Using the “Logical Operators” menu of the Segment Editor module, the skull segment was subtracted from the endocast segment, resulting in an endocast that precisely fit along the intracranial border (Figure 3D).

**Figure 4:**
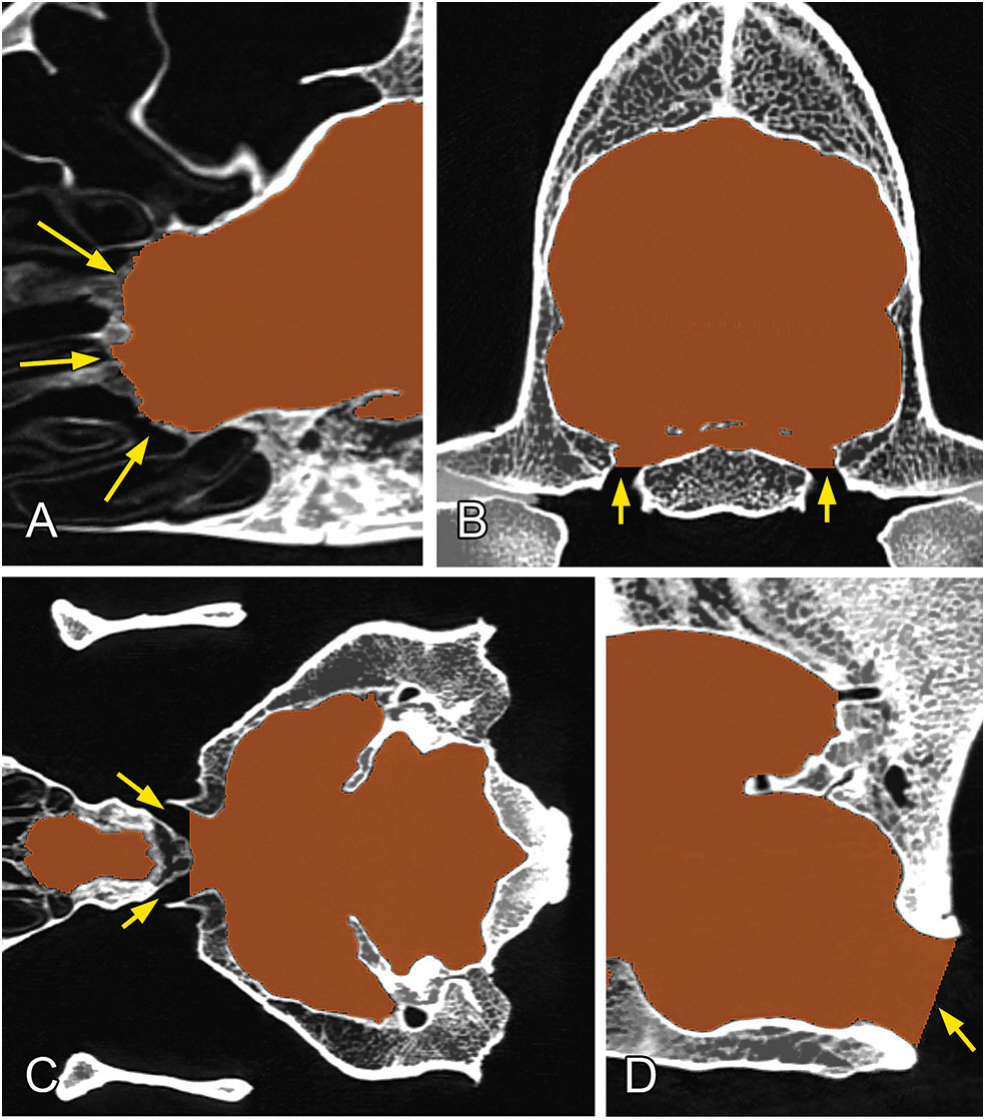
Closure of the different channels and openings of the neurocranium. **(A)** At the region of the lamina cribrosa (sagittal plane). **(B)** At the level of the foramen ovale (transverse plane). **(C)** At the level of the chiasma opticum (dorsal plane). **(D)** At the region of the foramen magnum (midsagittal plane). Yellow arrows show the channels/foramina.

The channels through which the different cranial nerves and vessels pass were closed manually by defining the planes to follow the same procedure on all of the skulls: the rostral end of the ethmoidal fossa was closed along the lamina cribrosa; the meatus acusticus internus and foramina ethmoidales were closed along the sagittal plane (Figure 4A); the canalis opticus, fissura orbitalis, foramen rotundum, foramen mastoideum, and canalis condylaris were closed along the transverse plane (Figure 4B); and the foramen ovale, foramen caroticum, canalis petrooccipitalis, foramen jugulare, and canalis nervi hypoglossi were closed along the dorsal plane (Figure 4C). Each channel was closed on the first slice where it did not have a direct connection with the endocranial space. The meatus temporalis was closed below and above the points where the channel was communicating with the neurocranial cavity. Due to the diversity of shapes of the foramen magnum across dog breeds, we used the following closure criteria for it: a caudally tilted transverse plane was set in a way to go through on the dorsal and ventral border of the foramen magnum at the midsagittal plane (Figure 4D). Using these procedures, we were able to produce a uniform segmentation process for all the skulls.

**Figure 5:**
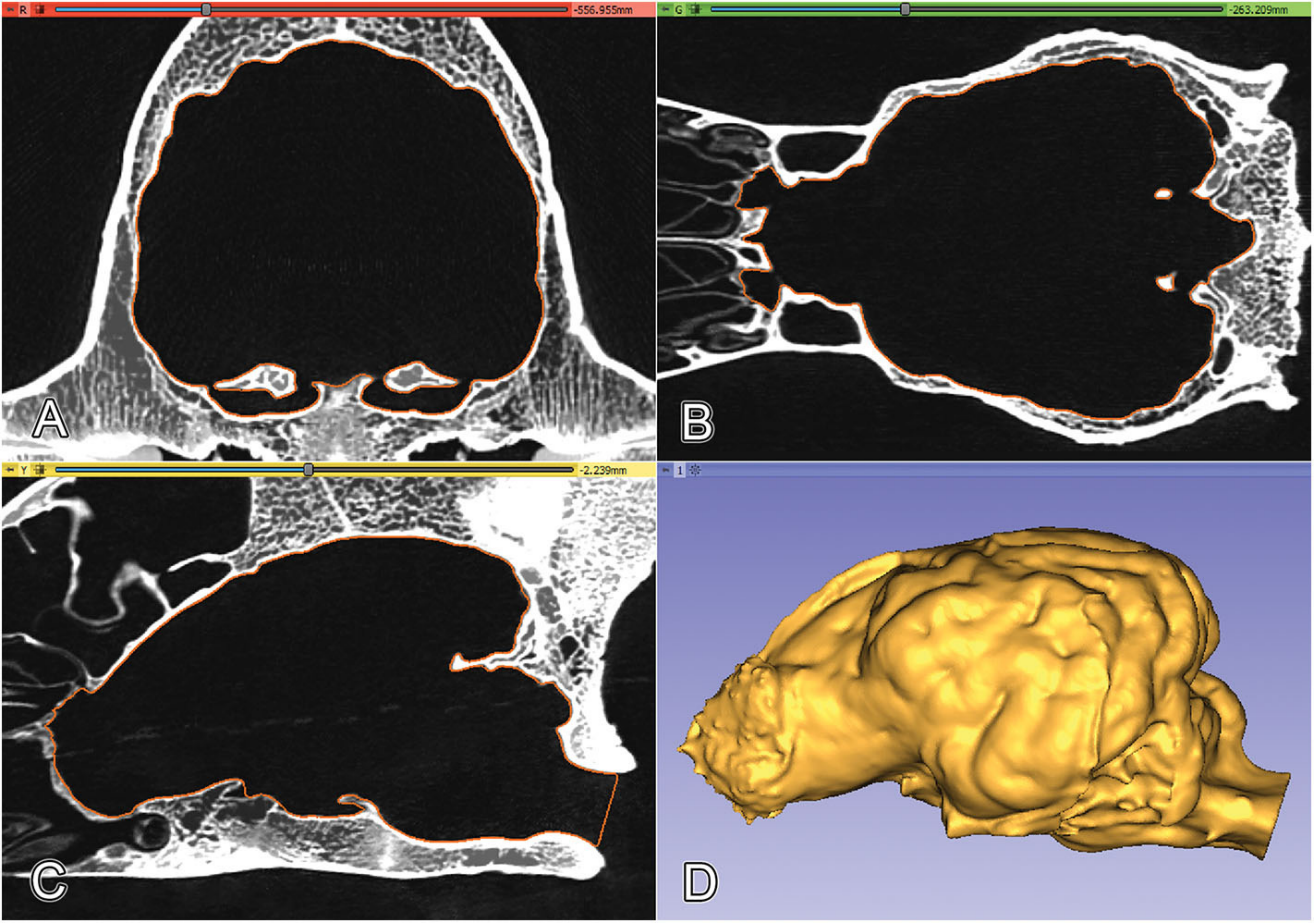
Projecting the surface mesh (marked with orange) onto the Saint Bernard's skull and checking its fitting on the orthogonal views. **(A)** Transverse view. **(B)** Dorsal view. **(C)** Sagittal view. **(D)** 3D model of the endocast of a Saint Bernard skull, left lateral view.

The endocast segment was then exported in a stereolithography (STL) format as a 3D surface mesh. Checking and refining the mesh was done with Autodesk Meshmixer (freeware, http://www.meshmixer.com); afterward, the STL file was imported into the 3D Slicer, where the surface mesh file was projected onto the orthogonal views. The complete endocranial volume was checked and controlled for its proper fitting (Figure 5). Anatomical terminology was used according to the 6th edition of the Nomina Anatomica Veterinaria (http://www.wava-amav.org/wava-documents.html).

#### Two- and Three-Dimensional Visualization

In order to show the different visualization possibilities which could be made based on this modeling technique, we created 2D standard images, then converted the 3D mesh into a 3D PDF format, which enables the users to rotate, colorize, annotate, or slice the model freely. Finally, using the selective laser sintering 3D-printing procedure with a Formiga P110 machine with PA2200 polyamide powder (EOS GmbH, München, Germany), some models were printed out and painted with acrylic dye to provide further visualization aid.

### Correlations Between Cranial and Endocranial Measurements

The analyses were carried on those digital endocasts which were created from the 28 skulls.

#### Calculating the Skull Index

**Figure 6:**
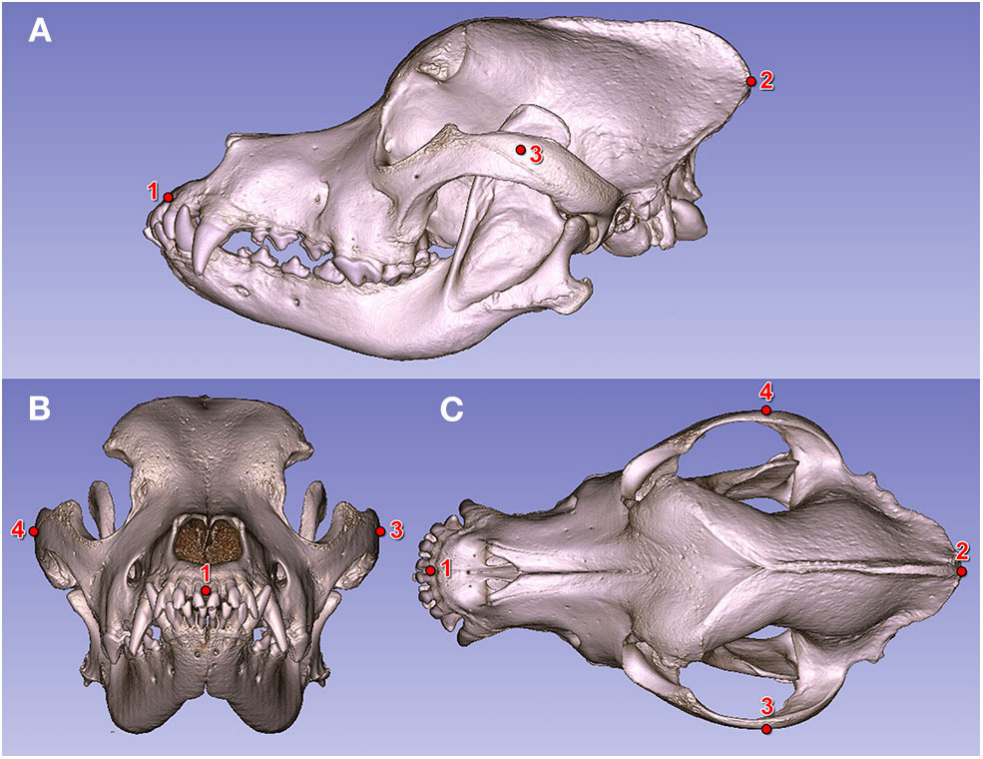
Fiducial markers on a Saint Bernard's skull (volume-rendered model). **(A)** Left lateral view. **(B)** Rostral view. **(C)** Dorsal view. (1) Prosthion. (2) Inion. (3) Widest point of the left zygomatic arch. (4) Widest point of the right zygomatic arch.

Measurements were done using the following landmarks: prosthion, inion (protuberantia occipitalis externa), and the widest point of the zygomatic arches (Figure 6). Skull indices (SI) were determined according to this formula:

$$\begin{array}{l} SI=\frac{skull\;width}{skull\;length}\;*\;100 \end{array}$$

where the skull width was measured between the widest point of the zygomatic arches and the skull length was measured between the prosthion and inion. Skulls having SI < 51 were classified as dolicocephalic, 51 ≤ SI < 59 were mesocephalic, and 59 ≤ SI considered to be brachycephalic types (41).

#### Calculating the Volume and the Surface Ratios

**Figure 7:**
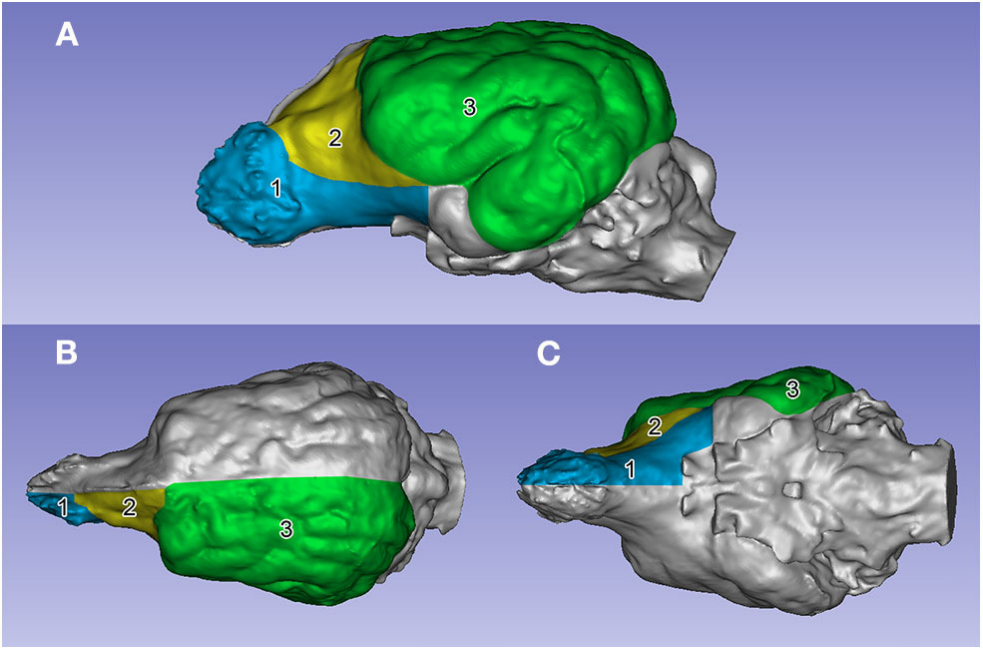
The segmented surface regions on the left side on an English setter's endocast. **(A)** Left lateral view. **(B)** Dorsal view. **(C)** Ventral view. (1) Prepiriform rhinencephalic area (in blue). (2) Prefrontal area (in yellow). (3) Non-prefrontal cerebral convexity area (in green).

Both the volume and the surface of a given STL model was automatically calculated and displayed with the 3D Slicer's Models module. To calculate the surface ratios, a sub-segmentation of the endocasts was performed. The surfaces of the endocasts were smoothed with Meshmixer (using a number 20 smoothing scale) to equalize the surface smoothness and decrease local inhomogeneities (e.g., imprintings of the vessels). The surface of each endocast was divided into subparts comprising the left and right prepiriform rhinencephalic, prefrontal, and non-prefrontal cerebral convexity areas (Figure 7). The prepiriform rhinencephalic area included the olfactory bulb, the olfactory trigone, and the olfactory peduncle, bordered by the lateral rhinal fissure and caudally at the transverse level of the orbital fissure. We did not include the piriform lobe due to the fact that no clear caudal boundary can be drawn on the parahippocampal gyrus, and because the greatest morphological alteration happens at the region of the rostral cranial fossa as a result of the shortening of the skull. The caudal border of the prefrontal area was drawn at the presylvian sulcus, and where the sulcus dorsally terminated, we drew a straight line toward the midline along the transverse plane. The rostral border of the prefrontal cortex was given by the olfactory bulb, next to the cribriform plate, and its ventral boundary was represented by the rostral part of the sulcus rhinalis lateralis. The non-prefrontal cerebral convexity area comprised the remaining cerebral surface on the lateral convexity up to the junction of the channel of the transverse sinus (thus comprising regions from the caudal part of the frontal lobe, and the visible parts of the temporal, parietal, and occipital lobes. This is why we used this non-conventional terminology to describe it during the analysis). The surface areas of the left and right sides from the same specimen were averaged as follows:

$$\begin{array}{l} mean_{prefrontal\text{\_}area}=\frac{prefronta{l\text{\_}area}_{left}+prefronta{l\text{\_}area}_{right}}{2} \end{array}$$

Thus, for each animal, we obtained three averaged surface values: prepiriform rhinencephalic (R), prefrontal (F), and non-prefrontal cerebral convexity (C) areas. For calculating area ratios (%), each average value was divided by the full hemicerebral surface, e.g.,

$$\begin{array}{l} R\%=\frac{R}{R+F+C} \end{array}$$

### Statistical Analysis

We used SPSSv25.0 for the analyses. The Spearman correlation was used for investigating the relationship between the endocast volumes, surface areas and proportions, and the skull index.

## Results

**Figure 8:**
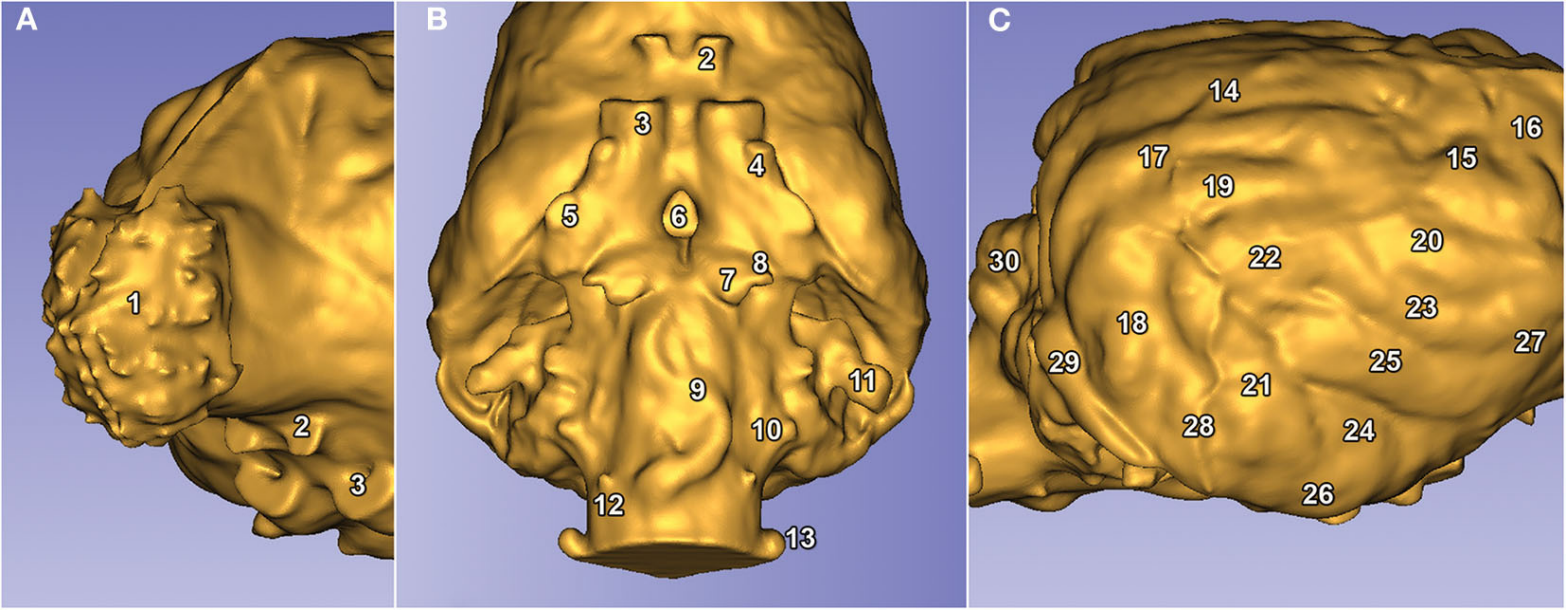
Visible structures on a Weimaraner's endocast. **(A)** Left rostro-lateral view. **(B)** Ventral view. **(C)** Right caudo-lateral view. The numbers represent the structures of the brain. (1) Bulbus olfactorius. (2) Canalis opticus. (3) Fissura orbitalis. (4) Foramen rotundum. (5) Foramen ovale. (6) Dorsum sellae. (7), Canalis petrooccipitalis. (8) Foramen caroticum. (9) Impression of the A. basilaris cerebri on the medulla oblongata. 10) Foramen jugulare. (11) Fossa subarcuata. (12) Canalis nervi hypoglossi. (13) Canalis condylaris. (14) Gyrus marginalis. (15) Gyrus postcruciatus. (16) Gyrus precruciatus. (17) Gyrus ectomarginalis. (18) Gyrus suprasylvius caudalis. (19) Gyrus suprasylvius medius. (20) Gyrus suprasylvius rostralis. (21) Gyrus ectosylvius caudalis. (22) Gyrus ectosylvius medius. (23) Gyrus ectosylvius rostralis. (24) Gyrus sylvius caudalis. (25) Gyrus sylvius rostralis. (26) Gyrus compositus caudalis. (27) Gyrus compositus rostralis. (28) Impression of the A. meningea media. (29) Sinus transversus. (30) Vermis cerebelli.

**Figure 9:**
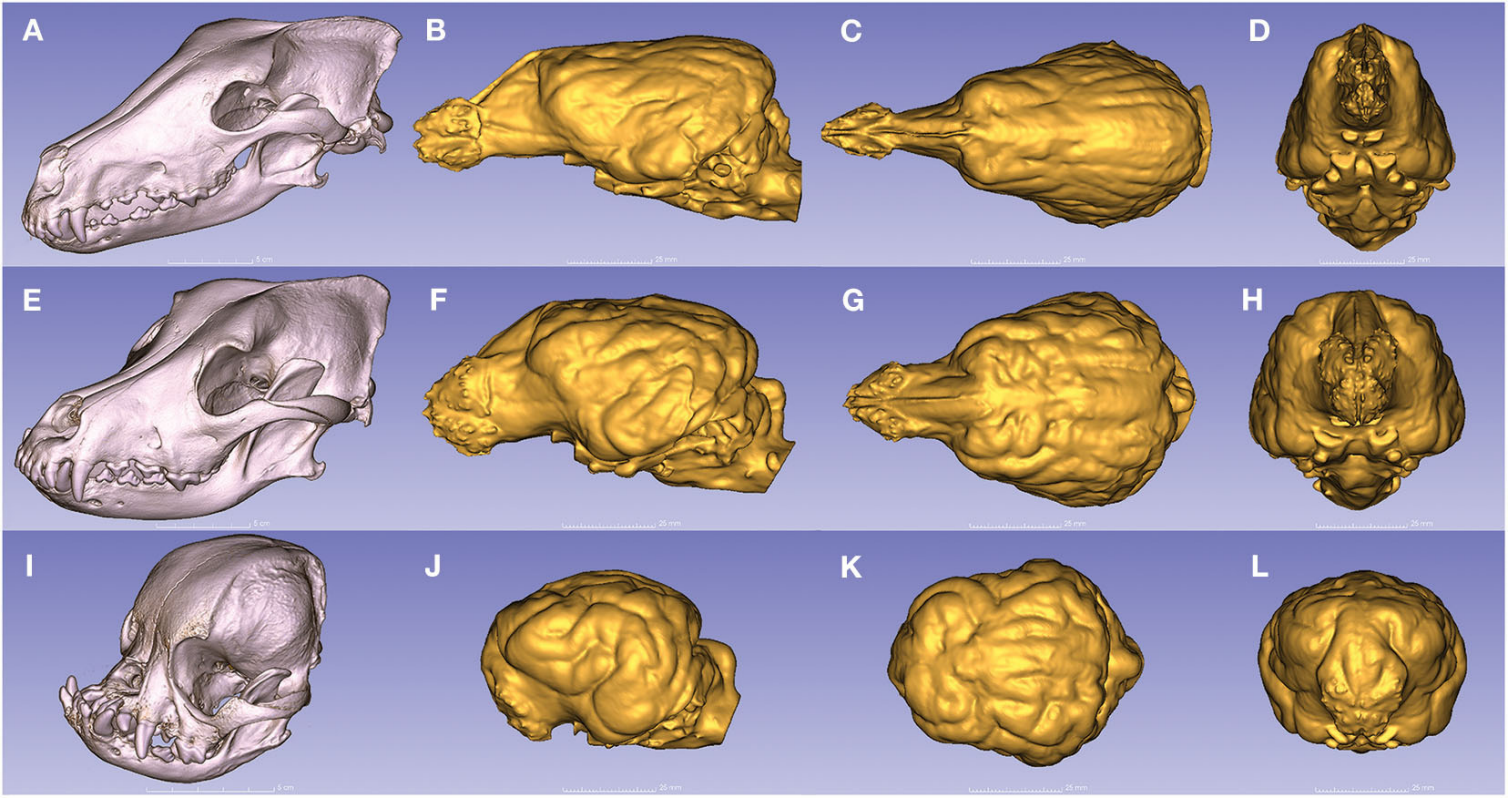
Endocasts from dogs with the three main head types. **(A)** The dolichocephalic skull of a borzoi. **(B–D)** Endocast of a borzoi. **(E)** The mesocephalic skull of a Rhodesian ridgeback. **(F–H)** Endocast of a Rhodesian ridgeback. **(I)** The brachycephalic skull of a pug. **(J–L)** Endocast of a pug. **(A,E,I)** Left rostro-lateral view. **(B,F,J)** Left lateral view. **(C,G,K)** Dorsal view. **(D,H,L)** Rostral view.

**Figure 10:**
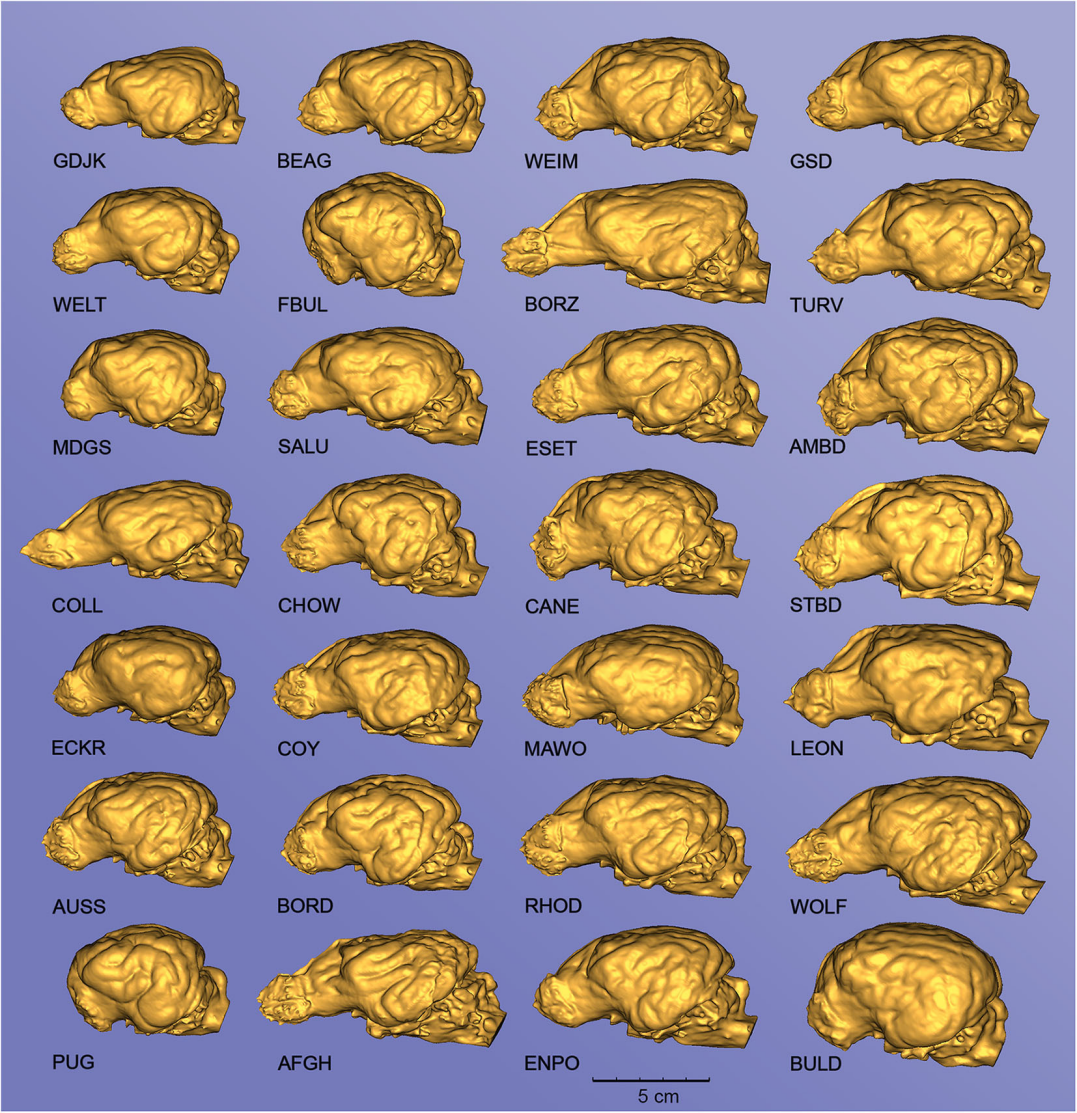
Endocasts from different canine specimens, showing the diversity of surface morphology. Left lateral view. GDJK, Golden jackal; BEAG, Beagle; WEIM, Weimaraner; GSD, German shepherd dog; WELT, Welsh terrier; FBUL, French bulldog; BORZ, Borzoi; TURV, Belgian shepherd dog (Tervueren); MDGS, Medium German spitz; SALU, Saluki; ESET, English setter; AMBD, American bulldog; COLL, Collie; CHOW, Chow chow; CANE, Cane corso; STBD, Saint Bernard; ECKR, English cocker spaniel; COY, Coyote; MAWO, Maned wolf; LEON, Leonberger; AUSS, Australian shepherd; BORD, Border collie; RHOD, Rhodesian ridgeback; WOLF, Gray wolf; PUG, Pug; AFGH, Afghan hound; ENPO, English pointer; BULD, English bulldog. The endocasts are grouped into columns according to an increasing total volume [thus the top left endocast (GJDK) represents the smallest endocast, and the bottom right (BULD) has the largest volume].

Due to the high-resolution CT scanning, the gyri and sulci can be properly distinguished on the surfaces of the endocast meshes (Figure 8), the casts of the olfactory bulbs also show the points where the olfactory nerves leave through the lamina cribrosa (Figure 8A/1), and even the impression of the basilar artery can be well-recognized on the ventral surface of the brainstem (Figure 8B/9). The 3D surface endocast models from the 28 skulls visualize the differences between meso-, brachy-, and dolichocephalic dogs' endocasts from three angels (Figure 9). We placed the 28 endocasts next to each other (in an increasing volume order), showing the differences in their sizes and shapes (Figure 10).

**Figure 11:**
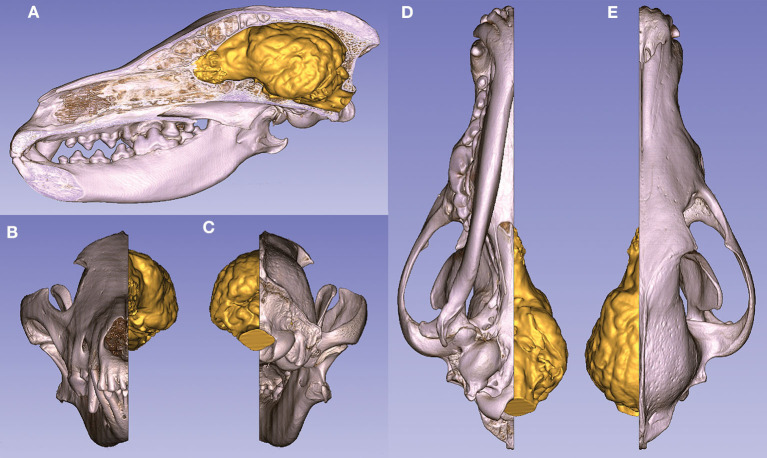
Volume-rendered skull model (with a midsagittal section) and the endocast of a gray wolf. **(A)** Left lateral view. **(B)** Rostral view. **(C)** Caudal view. **(D)** Ventral view. **(E)** Dorsal view.

Two models can be interactively viewed as PDF documents, where rotation, slicing, annotation, measurements, and changes in lighting and opacity can be freely performed. Annotations were made in these 3D PDF documents to show the main gyri, sulci, and openings of the brain on these two endocasts, belonging to a Weimaraner and a French bulldog (https://figshare.com/articles/_/12363596). The volume rendered and the surface models can also be visualized in the same coordinate system to show their relationship (Figure 11).

**Figure 12:**
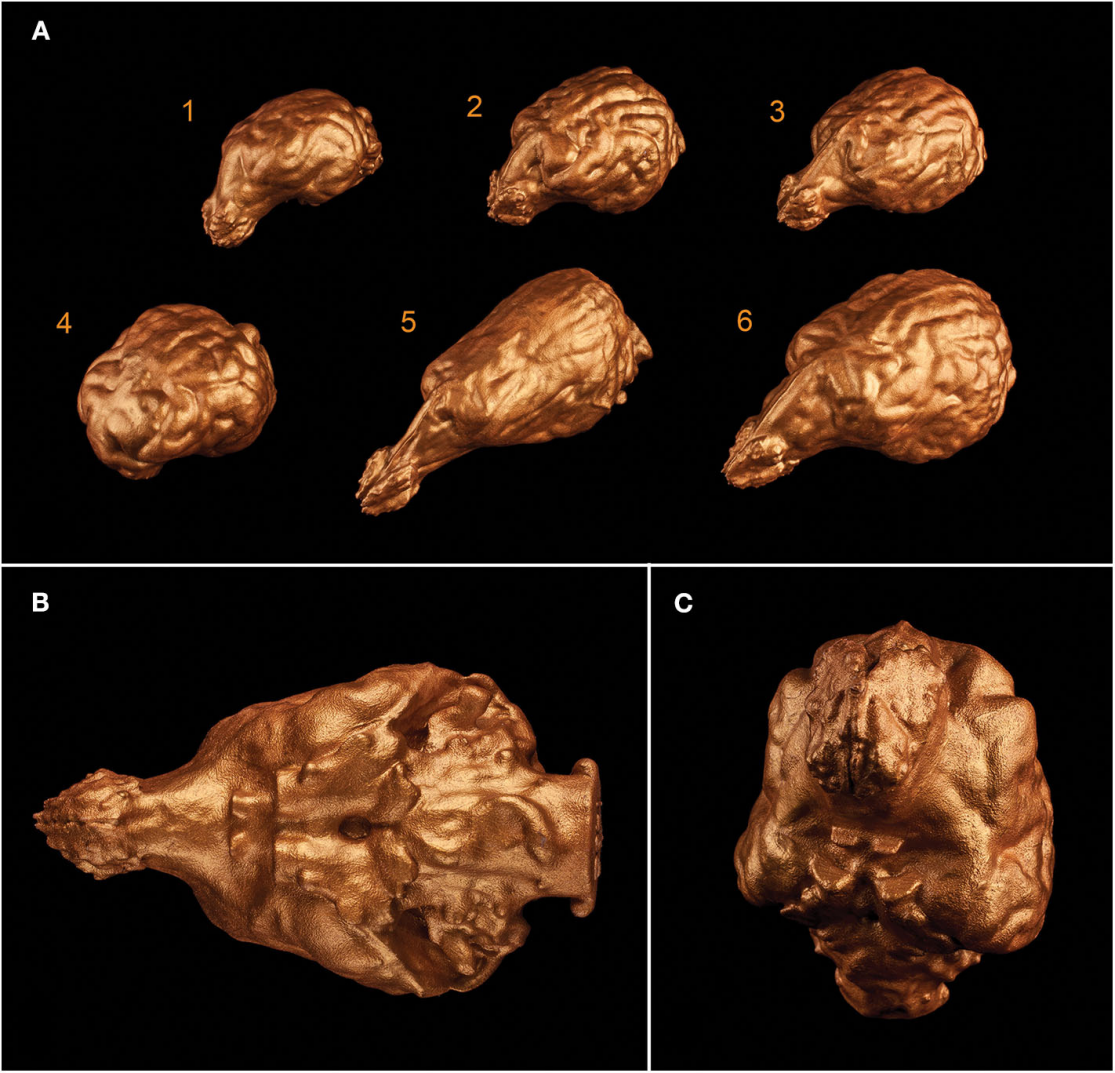
3D prints of different endocasts. **(A)** Endocast of a Welsh terrier (1), a border collie (2), a Weimaraner (3), a pug (4), a borzoi (5), and a gray wolf (6). **(B)** Ventral aspect of the endocast of a Weimaraner. **(C)** Rostroventral aspect of the endocast of a border collie.

**Figure 13:**
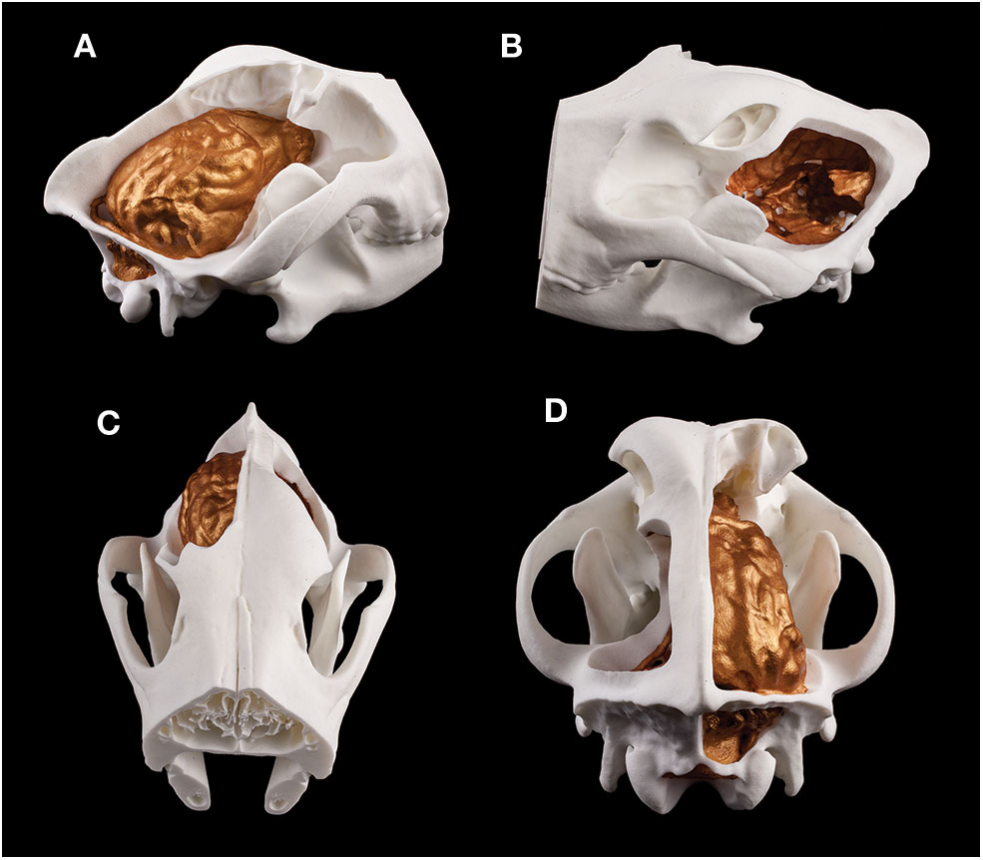
Composite 3D-printed model of a skull and the endocast of a Weimaraner. **(A)** Right lateral view. **(B)** Left lateral view. **(C)** Rostrodorsal view. **(D)** Caudodorsal view.

3D printing gave us a unique possibility to examine the endocasts in their actual size and provided an in-hand comparison between different breeds (Figure 12). In the case of one sample, the 3D model of the skull was digitally opened on the left side to see inside the neurocranial cavity, while on the right side the endocast was mounted onto the bone by removing the outer osseous lamina and diploe from the occipital, parietal, temporal, and frontal bones together with opening the frontal sinuses in order to show the position of the brain inside the skull on the 3D print (Figure 13).

**Figure 14:**
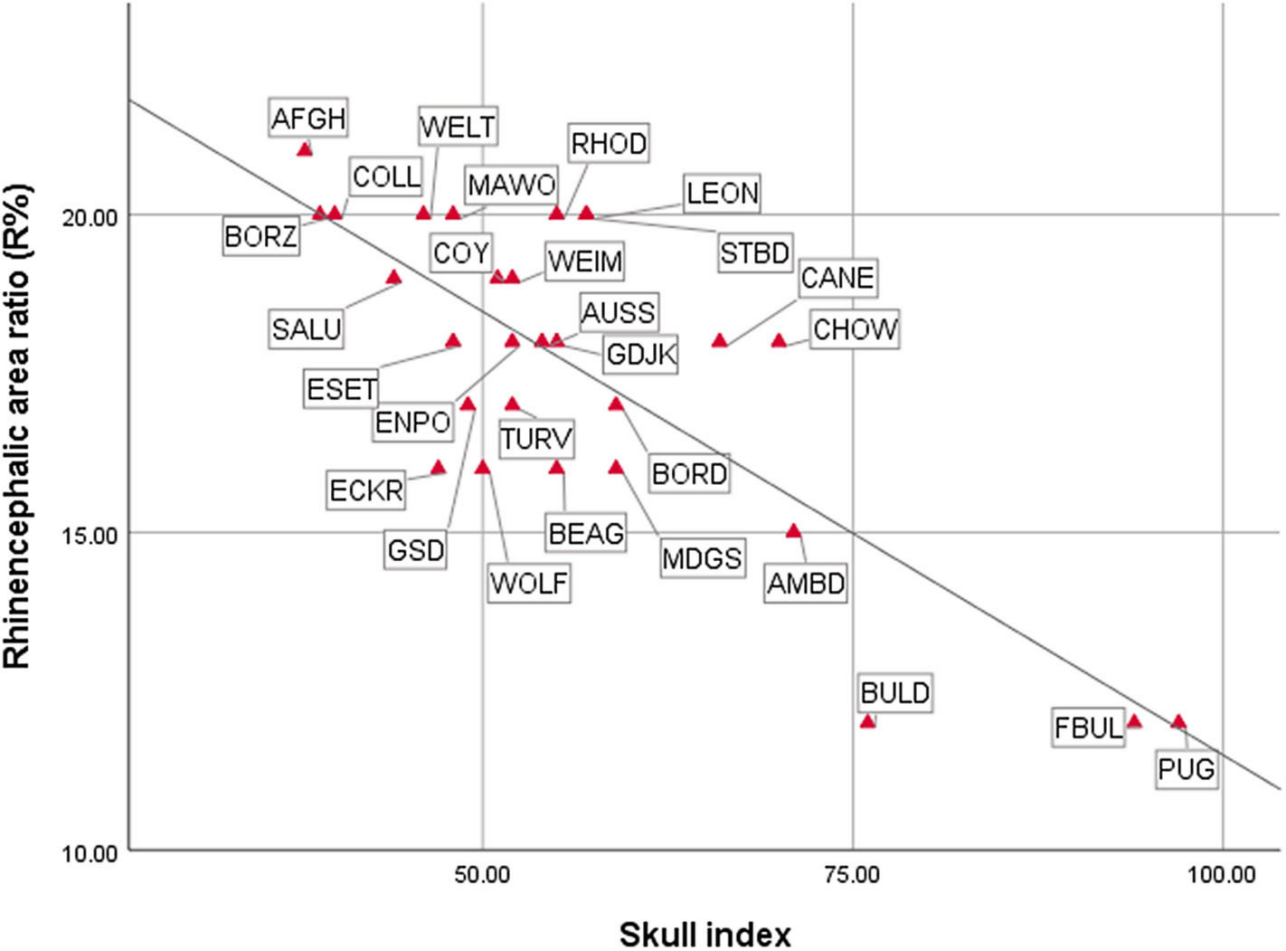
Correlations between cranial and endocranial measurements. For breed/species name abbreviations, see Figure 10.

As expected, the endocast volumes, averaged endocast areas, skull length, and skull width positively correlated with each other, i.e., larger headed dogs had larger endocast surfaces (for statistical details, see Table 1). The specific endocast area ratios (%) correlated differently with skull length and width. *R*% (prepiriform rhinencephalic ratio) and *F*% (prefrontal ratio) correlated positively with skull length, and negatively with *C*% (non-prefrontal cerebral convexity ratio), i.e., longer-headed dogs had proportionally larger rhinencephalic and smaller non-prefrontal cerebral areas. Of course, based on the endocasting method we could only assess the visible surface of these areas (e.g., a part from the prefrontal region is hidden by the olfactory bulb), and could not measure actual volumes. Skull width and endocast volume did not correlate with any endocast area ratio; thus, neither skull width nor endocast volume affects endocast area ratios. In harmony with the findings with skull length, the skull index correlated negatively with *R*% (Figure 14), did not correlate with *F*%, and correlated positively with *C*% (Table 1); thus, dolichocephalic dogs had proportionally larger rhinencephalic and non-prefrontal cerebral convexity areas than brachycephalic dogs. Dogs with the highest skull index (the French bulldog and pug) substantially differed from all other dogs in their exceptionally small prepiriform rhinencephalic area.

**Table 1:** Spearman's correlations between skull and endocast parameters.

	**Volume**	**Skull length**	**Skull width**	**Skull index**	**Avg. R**	**Avg. F**	**Avg. C**	***R*%**	***F*%**
Skull length	0.694^**^								
Skull width	0.839^**^	0.513^**^							
Skull index	0.131	−0.423^*^	0.486^**^						
Avg. R	0.808^**^	0.874^**^	0.556^**^	−0.297					
Avg. F	0.751^**^	0.749^**^	0.612^**^	−0.053	0.718^**^				
Avg. C	0.955^**^	0.548^**^	0.807^**^	0.258	0.688^**^	0.667^**^			
*R*%	0.007	0.543^**^	−0.084	−0.595^**^	0.526^**^	0.156	−0.183		
*F*%	0.059	0.523^**^	0.042	−0.357	0.331	0.605^**^	−0.048	0.440^*^	
*C*%	−0.084	−0.631^**^	0.020	0.607^**^	−0.567^**^	−0.425^*^	0.097	−0.890^**^	−0.735^**^

* *p < 0.05*,

** *p < 0.01*.

*R, prepiriform rhinencephalic area; F, prefrontal area; C, non-prefrontal cerebral convexity area; avg., average*.

## Discussion

In this study, we digitized 28 canine skulls using computed tomography, created virtual endocast models in both 2D images, 3D PDFs, and 3D prints. We presented endocasts with a higher spatial resolution (<0.5 mm) than standard medical imaging (which is usually >0.5 mm); therefore, they can be used to visualize in detail the endocranial space in its entirety. The surface morphology, placement of the channels for the cranial nerves, and even traces of the vascular system can be effectively shown with this technique. We also presented the casts from different directions and in comparison with each other, and also as 3D PDF files, which allows the viewer to interact with the content (e.g., hide or show different parts of the 3D model, turn in any angle, make annotations or measurements, or look inside the model). Digital models can also be 3D printed, which gives an additional tool for evaluation and education, as the models can be replicated in the required number, detail, and size. It should be noted that the CT-based segmentation of an endocast from a skull cannot provide those details which would derive from an MRI-examination which visualizes the brain itself [thus, using an actual brain during structural imaging results in more detailed segmentation and region-specific volumetric measurements can be also performed (42, 43)]. Our intention was to show that creating an endocranial cast from a CT-dataset can also offer a good alternative (if only the skull is available) to study the macroscopic surface anatomy. We demonstrated different surface structures of the brain (e.g., gyri, sulci, and foramina) on 3D models. The casts clearly delineated the shape of the olfactory bulbs, and even the impressions of the major arteries are recognizable on the surface (Figure 8). To our knowledge, canine digital endocranial casts have not been created before in this number regarding different breeds and wild species. This approach might help to characterize neuroanatomical variation resulting from domestication processes by comparing endocasts of dogs and other canids. As wild forms changed through the course of domestication, these changes in behavior and behavioral patterns could be linked to changes in the size and proportions of different brain regions (34). It is unclear whether the relation between brain volume and executive function reflects a broad-scale evolutionary phenomenon or a unique consequence of primate brain evolution. Primates show associations between brain volume and differences in some aspects of cognition (44). The extraordinary degree of intraspecific morphological variation in domesticated dogs offers a unique opportunity to study this phenomenon. High definition endocasts could help provide more detailed neuroanatomical measures that could be used to more precisely link neuroanatomy to cognitive abilities (i.e., social cue following).

It has been argued that the mammalian brain, particularly its size, is affected by natural selection and influences particular behavioral capacities. Based on comparative data it was demonstrated that mosaic changes are an important factor in brain structure and evolution, suggesting that brain evolution involved complex relationships among the individual brain components (45). The frontal cortex structure and development can differ substantially between taxa. For example in primate, but not carnivore, frontal cortex hyperscales relative to the rest of neocortex and the rest of the brain (46). Moreover, researchers examined a group of birds in the corvid family and found that some species had a larger relative hippocampus than others (47). Interestingly, the species with a bigger hippocampus shows better performance on spatial memory tasks. It was also shown that the size of the cerebellum increases throughout the evolution of apes and even humans (48). This difference suggests that the cerebellar specialization could be involved in the evolution of humans' advanced technological capacities and even as a preadaptation for language. Dogs have been exposed to strong selective pressure and the result of this artificial selection brought to different breeds with a variety of working roles. For instance, it has been suggested that cooperative working breeds have also a higher proficiency when it comes to following human social cues (49, 50). In an MRI-based analysis, it was found that neuroanatomy co-varies together with behavioral specializations (i.e., sight hunting, scent hunting, guarding, and companionship) (38). Another study revealed the existence of relationships between body size, skull shape, and behavior among dog breeds (51). These results reinforce the assumption that dogs' morphotypes (including the cephalic index, that has an effect in the brain shape) can be associated with particular behavioral profiles (e.g., grooming, chasing, aggression). As increase in the brain size was proved to be positively linked with the executive function (37), dogs might be considered a powerful model for studying evolutionary links between cognition and neuroanatomy. Hence, we argue that the study of detailed endocasts and the study of the neuroanatomical variations could give helpful aid to pinpoint and document furthermore the differences between breed groups or working roles beside the MRI studies.

Our results could give an aid for visualization and educational purposes. We created and visualized endocasts of several dog breeds and based on these endocasts, detailed morphometric analyses and interspecific comparisons (regarding volume, surface, or ratio) can be performed in the future. The analyses of the endocast area proportions showed that the prepiriform rhinencephalic area significantly decreased with the larger skull index. The limitation of the study is the relatively low number of skulls and that only one individual represents a breed or a wild species, without information whether this individual is a typical representative of its breed/species or not. However, we presented a wide range of skulls with different cephalic index, and showed the diversity of the endocasts' (and based on this, the brains') morphology (e.g., with Figure 10). Further studies with more individuals should validate the representativity of ours skulls and investigate how the reduced rhinencephalic area is linked to the relatively poor performance of brachycephalic dogs in olfactory search tasks (52). By assessing the surface one should know that only the external surface of the brain is visible on an endocast, and some parts (like the prefrontal lobe) are partly hidden due to the overlap of adjacent structures. Consequently, when it is possible, an MRI-based volumetric assessment is recommended. Despite that fact, the surface analysis using the endocasts could give a good tool to compare the degree of modification (concerning the changes in the skull shape) of the brain, especially when only the skull of an animal (or its DICOM data) is available. Brachycephalic dogs typically have a smaller angle between the olfactory bulb fissure and the baseline of the cranial cavity, causing the ethmoid turbinates to be more ventrally oriented and protrude into the nasal airways (53). This protrusion also contributes to health issues, such as brachycephalic obstructive airway syndrome (54). A study reported similar findings based on paramedian sagittal magnetic resonance imaging slices of canine brains, i.e., brachycephalic brains are rounded and shortened in the anterior-posterior plane with a shifted olfactory lobe (55). We also demonstrated that the length of the skull has a significant effect on the endocast area ratios, whereas the skull width has no effect on the measured areas.

The digitalization process with structural imaging for making virtual endocasts does have a substantial cost (e.g., paying for the CT scanning time), and the 3D modeling work also requires knowledge of the specific software used during the segmentation and mesh-refine steps. Despite that, we believe that its benefits are well worth these investments, as described in the introduction. In conclusion, the digital canine endocranial casts we created can be useful for both educational and comparative purposes, considering the growing interest in the virtual-, augmented-, and mixed reality fields which require high resolution surface models.

## Data Availability Statement

A portion of the datasets analyzed in this study are available through Figshare (https://doi.org/10.6084/m9.figshare.12363596). The rest of the data analyzed in this study are available in the article and Supplementary Material.

## Ethics Statement

Ethical review and approval was not required for the animal study because we used only the skulls of different canines species from a private collection for the computed tomography (CT) examination.

## Author Contributions

KC: conceptualization. KC, AS, ÖP, TC, and EK: methodology, writing—review, and editing. KC, AS, and EK: data curation and writing—original draft. KC and EK: formal analysis. All authors contributed to the article and approved the submitted version.

## Conflict of Interest

The authors declare that the research was conducted in the absence of any commercial or financial relationships that could be construed as a potential conflict of interest.
